# Root Morphology and Rhizosphere Characteristics Are Related to Salt Tolerance of *Suaeda salsa* and *Beta vulgaris* L.

**DOI:** 10.3389/fpls.2021.677767

**Published:** 2021-06-21

**Authors:** Shoule Wang, Zhenyong Zhao, Shaoqing Ge, Bin Peng, Ke Zhang, Mingfang Hu, Wenxuan Mai, Changyan Tian

**Affiliations:** ^1^State Key Laboratory of Desert and Oasis Ecology, Xinjiang Institute of Ecology and Geography, Chinese Academy of Sciences, Urumqi, China; ^2^University of Chinese Academy of Sciences, Beijing, China

**Keywords:** *Suaeda salsa*, *Beta vulgaris*, root morphology, rhizosphere, salinity, nutrition

## Abstract

Halophytes are capable of resisting salinity, and their root system is the part in direct contact with the saline soil environment. The aim of this study was to compare the responses of root morphology and rhizosphere characteristics to salinity between a halophyte, *Suaeda salsa* (suaeda), and a glycophyte, *Beta vulgaris* L. (sugar beet). The soil salt content was set to four levels (0.7, 1.2, 1.7, and 2.7%) by NaCl-treated plants. We investigated the soil pH, EC, nutrients and soil, plant ion (Na^+^, Cl^−^, K^+^, and Mg^2+^) concentration to evaluate the rhizospheric processes, and salt tolerance of suaeda by the root mat method. The highest biomass was in the 1.2% salt level for suaeda and in the 0.7% salt level for sugar beet. The root length and root surface area of suaeda showed similar trends to biomass, but the root diameter decreased by 11.5–17.9% with higher salinity. The Na^+^, Cl^−^, and K^+^ accumulations in the shoot of suaeda displayed higher than that in sugar beet, while the Mg^2+^ accumulation was lower in suaeda than that in sugar beet. High salinity resulted in increased pH and EC values in the rhizosphere for suaeda, but lower values of these parameters for sugar beet. Under high salinity, the Olsen phosphorus content was 0.50 g·kg^−1^ and 0.99 g·kg^−1^ higher in the rhizosphere than in the non-rhizosphere for suaeda and sugar beet. We concluded that the two species [halophyte, *Suaeda salsa* (suaeda), and a glycophyte, *B. vulgaris* L. (sugar beet)] showed diverse approaches for nutrient absorption under salinity stress. Suaeda altered its root morphology (smaller root diameter and longer roots) under salt stress to increase the root surface area, while sugar beet activated rhizospheric processes to take up more nutrients.

## Introduction

Halophytes are plants that can complete their entire life history on highly saline soils. These plants have a series of adaptive strategies that have arisen during their coordinated evolution with the environment (Flowers, 2004; Shabala et al., 2014; Chaudhary et al., 2018; Qiao et al., 2018). Several studies have shown that halophytes have special mechanisms to resist and alleviate salinity stress (Bennett et al., 2013; Yuan et al., 2016; Dassanayake and Larkin, 2017; Etesami and Beattie, 2018; Ma et al., 2019). Special structures and ion transport systems alleviate the negative effects of salinity and are important adaptations to enhance plant survival under stress (Shabala et al., 2015; Belghith et al., 2018; Nikalje et al., 2018). Osmotic substances are produced inside the cell to reduce water potential and accelerate uptake of water from the outside. Under salinity, the H^+^-ATPase activities are ameliorated to establish the proton concentration gradient across the membrane, and Na^+^/H^+^ antiporter (responsible for counter-transport of Na^+^ and H^+^ across membranes) accelerates Na^+^ compartmentalization into vacuoles (Song et al., 2005; Lv et al., 2012; Wang et al., 2019). Ma et al. (2019) always found that variation in cell size with salinity mostly impacted the growth of euhalophytes and their tissue hydration. To date, despite the large researches conducted on halophytes and focused on the above-ground tissue (Song et al., 2009; Belkheiri and Mulas, 2013; Ma et al., 2019), the comprehension of the plant survival strategy under salinity still remains inadequate, especially in terms of the responses of root morphology and rhizosphere characteristics to high salinity.

Roots are the organ with the most direct exposure to salinity, and their structure and function are one of the main features in the life cycle of halophytes (Shabala et al., 2015; González-Orenga et al., 2020). The root systems of many species exhibit morphological plasticity, which increases the ratio of fine roots and improves the contact area so that they can absorb more nutrients in low-nutrient soils (Zhang et al., 2010; Li et al., 2014; Mai et al., 2018). Like nutrients, various salts show great spatial and temporal variations in their concentrations in saline-alkali soil (Chen et al., 2019). The patterns of salt distribution in the soil strongly affect the spatial distribution of plants, as well as the configuration, length, and size of their root systems. High salinity affects their root morphological traits and restricts the growth of halophytes (Song, 2009; González-Orenga et al., 2020). The roots of halophytes responded directly to sodium (Na^+^) and chlorine (Cl^−^) ions are obviously affected by other soil characteristics. This affects plants’ demand for nutrients in certain habitats (Chen et al., 2017). Previous studies have detected increased organic matter content and higher enzyme activity of the soil after planting halophytes (Yi and Zhang, 2011; Yang et al., 2016). Furthermore, in the rhizosphere, changes in the microbial population and acceleration of soil nutrient leaching occur under salt stress (Ilangumaran and Smith, 2017; Radhakrishnan and Baek, 2017). Some studies found that a heterogeneous group of bacteria benefit greatly from the rhizosphere and promote plant growth (Bashan et al., 2000; Jha et al., 2011; Ilangumaran and Smith, 2017; Etesamia and Maheshwarib, 2018; Komaresoflaa et al., 2019). Thus, plants, microorganisms, and soil make up a complex network through which materials are delivered and transferred into the micro-ecological environment of the rhizosphere (Santos et al., 2015; Qin et al., 2017; Langarica-Fuentes et al., 2018). The root exudates of halophytes are another significant factor in salt tolerance and nutrient-use efficiency (Ben Hassine et al., 2009). This suggested that rhizosphere processes also play important roles in the tolerance of halophytes to salinity, where utilizing various physical and chemical mechanisms, such as alteration in root architecture, secretion of organic acids, and production of extracellular enzymes, increase nutrient acquisition efficiency under high salinity (Wang et al., 2020). However, what are the effects of salt stress on rhizosphere characteristics and how the root morphology of halophytes responds to salinization still remain unclear. The clarification of these issues will help us to better understand salt tolerance mechanisms and strategies to absorb nutrients from highly saline soils.

*Suaeda salsa* (suaeda, in the family *Chenopodiaceae*) is an annual euhalophyte that grows in saline soils, and the species possesses the capacity in understanding 200–400 mM NaCl and has been ideal species salt tolerance studies (Song, 2009; Liu et al., 2018; Ma et al., 2019). The salt content affects the soil pH in pot experiments (Xian et al., 2019). Thus, we included *B. vulgaris* L., which is relatively salt tolerant, to maintain experimental consistency. *B. vulgaris* (also in the family *Chenopodiaceae*) is an annual glycophyte that has strategy in response to salt stress to a certain extent (Vitali et al., 2021). In this study, therefore, we conducted a pot experiment with two species: the euhalophyte, *Suaeda salsa*, and the glycophyte, *B. vulgaris* L. The objectives of the present study were to: (1) evaluate the effect of salt stress on root morphology (root length and surface area) and (2) determine changes in rhizosphere processes in response to salt stress.

## Materials and Methods

### Field Soil

The experiment was conducted in a greenhouse under a 14-h light/10-h dark photoperiod (25°C days and 20°C nights). The saline soil was collected from an experimental station in Changji, Xinjiang Province, China (44°09'59" N, 87°04'56"E). The soil was sampled in the depth 0–20 cm and then air-dried, passed through a 2-mm sieve. The basic physicochemical properties of the experimental soil were as follows: pH 7.64, EC 1.45 mS, total salt 0.70%, available nitrogen (N, NO_3_^−^, and NH_4_^+^) 33.68 mg·kg^−1^, and Olsen phosphorus (Olsen-P) 4.62 mg·kg^−1^.

### Experimental Setup

**Figure 1:**
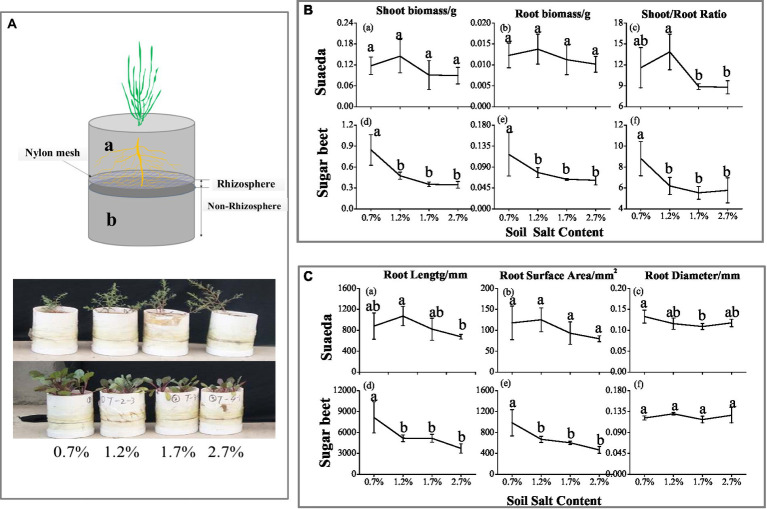
**(A)** Schematic diagram of the root system. Growing tube was divided into upper 10-cm height **(A-a)** and lower 5-cm depth **(A-b)**, separated by 30-μm nylon mesh. After harvesting, the lower root system was divided into eight parts (0–1 mm, 1–2 mm, 2–3 mm, 3–5 mm, 5–10 mm, 10–20 mm, 20–30 mm, and 30–50 mm). **(B)** Shoot biomass **(a, d)**, root biomass **(b, e)**, and top root ratio **(c, f)** of suaeda and sugar beet under different soil salinity treatments. **(C)** Root length **(a, d)**, root surface area **(b, e)**, and root diameter **(c, f)** of suaeda and sugar beet under different soil salinity treatments.

In this study, the root mat method was used to distinguish rhizosphere soil (0–3 mm) from non-rhizosphere soil (Figure 1A). This system was established in PVC tubes with an inner diameter of 10 cm and height of 15 cm. In the tubes, the upper layer (10 cm) was separated from the lower layer (5 cm) by 30-μm nylon mesh. This allowed us to collect soil samples at defined distances from the roots by slicing the soil parallel to the planar mat of roots developed on the mesh surface (Hinsinger and Gilkes, 1996). Based on the salt content of the original soil, we established four salinity treatments (0.7, 1.2, 1.7, and 2.7%) by adding NaCl dissolved in water.

Each pot was filled with 1.5 kg air-dried soil. To ensure adequate nutrient supply for plant growth, the soil was also fertilized with basal nutrients as follows (mg per pot): KH_2_PO_4_, 630 mg, and urea, 711 mg. Then, 20 suaeda seeds or 6 sugar beet seeds were sown into each pot and grown to 4-cm height before thinning. The NaCl was dissolved in water and added to the pot every 3 days for a total five times. The weighing method was used to supply water to field capacity (20%, w/w).

After 45 days of growth, the shoots and roots of suaeda and sugar beet were separated and collected for biomass measurements. The roots were washed in tap water. The root and shoot samples were killed by heating at 105°C (30 min) and then dried at 65°C for 48 h to calculate the biomass.

### Sampling Analysis

The soil in the “b” portion of the tube was divided into eight parts from top to bottom, and each part was dried and passed through a 2-mm sieve (Figure 1A–b). Soil pH and EC were measured in 1: 5 mixtures (10 g soil and 50 ml water), and soil available nitrogen (N) was extracted with 0.01 mol·L^−1^ CaCl_2_ (5 g soil and 50 ml solution), and soil available phosphorus (Olsen-P) was extracted with 0.5 mol·L^−1^ NaHCO_3_ (2.5 g soil and 50 ml solution; Bao, 2000). Diluted extracts were analyzed for Na^+^, K^+^, and Mg^2+^ (Flame Photometer, 735 ICP-OES, Agilent Technologies, United States) and Cl^−^ (AgNO_3_ titration method; Bao, 2000).

Roots were washed with deionized water and then scanned with an EPSON root scanner at 400 dots per inch resolution (Epson Expression 1600 pro, Model EU-35, Epson, Tokyo, Japan). The total root length was calculated using root analysis software DT-scan version 1.0 (Delta-T Devices, Burwell, United Kingdom). After scanning, the roots were killed by heating to 105°C (30 min) and then dried at 65°C for 48 h to calculate the biomass.

### Statistical Analysis

Analysis of variance (ANOVA, one-way) was conducted to detect differences in root morphological parameters among treatments using SPSS statistical software (SPSS version 19.0, IBM SPSS Inc., Chicago, IL, United States) and R software (version 4.0.3-win). Furthermore, the differences in soil ions content between the rhizosphere and the non-rhizosphere, and the shoot ions content between suaeda and sugar beet under different salinity were investigated. Significant differences among means were separated by the (LSD) test at the *p* < 0.05 probability level, and it was reflected by different letters in the figures by Duncan’s test. Using smooth fitting, we investigated the spatial variations of the pH and nutrients to explore the potential effects of salinity on halophyte rhizosphere processes. To clarify these effects, we compared parameters and responses between 0.7 and 2.7% salt levels.

## Results

### Plant Biomass and Root Morphology

The root biomass and shoot biomass of sugar beet decreased as the salt content in soil increased (Figure 1B–d,e), while the highest biomass of suaeda was in the 1.2% salt level (Figure 1B–a,b). And the shoot/root ratio in suaeda showed higher in the 1.2% salt level (Figure 1B–c,f), while it displayed decreasing trend in sugar beet. The shoot/root ratio was significantly higher in suaeda than that in sugar beet (*p* < 0.05). The variations in root morphological characteristics of suaeda and sugar beet are shown in Figure 1C. The root length and surface area always preformed higher in the 1.2% salt level, and a decreasing trend in sugar beet (Figure 1C–a,b,d,e). However, the root diameter of suaeda significantly decreased by 11.5–17.9% with salinity in soil, while it displayed non-significant (Figure 1C–c,f).

### Soil pH and Electrical Conductivity

**Figure 2:**
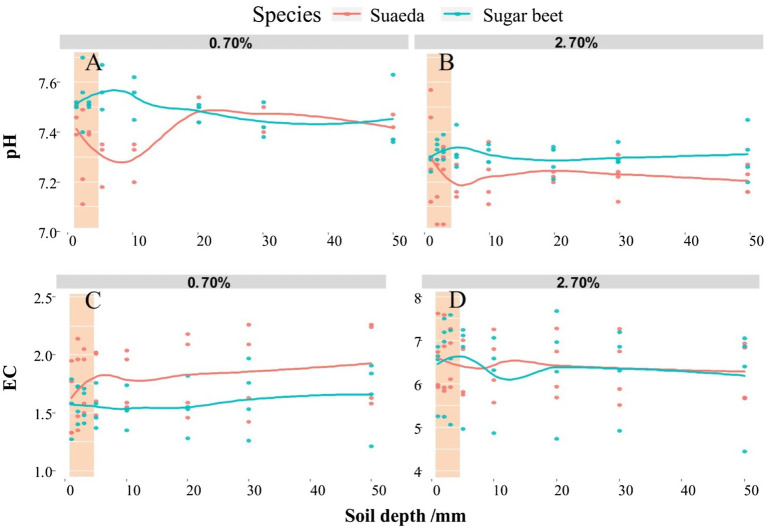
Spatial variations in pH **(A,B)** and electrical conductivity (EC; **C,D**) in different soil layers of the root systems of suaeda and sugar beet. Columnar shadow represents pH and EC in the rhizosphere.

We determined the pH and EC values in the rhizosphere of sugar beet and suaeda under 0.7 and 2.7% salt levels (Figure 2). For both species, the soil pH was lower in the 2.7% salt level than in the 0.7% salt level. In the 2.7% salt level, for suaeda, the pH and EC value were 0.12 and 0.22 higher in rhizosphere soil than in non-rhizosphere soil; for sugar beet, the pH and EC value were 0.05 and 0.22 lower in rhizosphere soil than in non-rhizosphere soil.

### Spatial Variations in Available N and Olsen-P Contents

**Figure 3:**
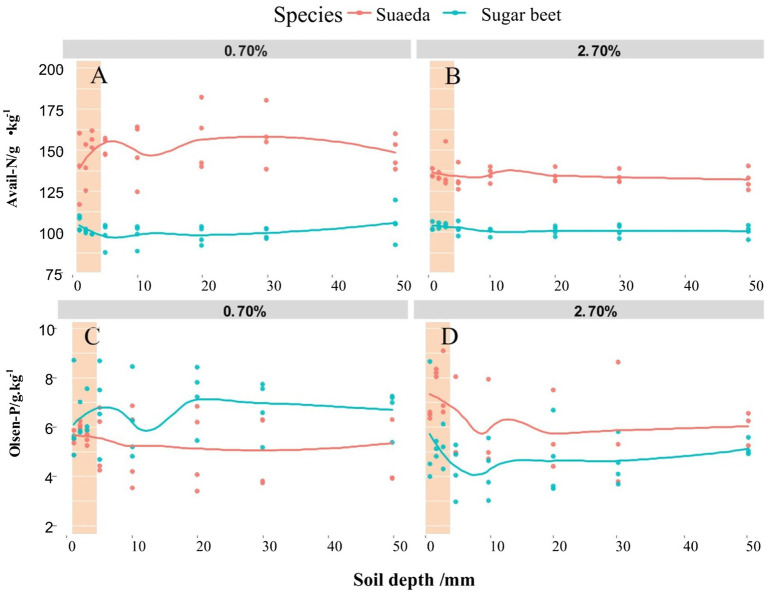
Spatial variations in available N **(A,B)** and Olsen-P **(C,D)** contents in different soil layers of the root system of suaeda and sugar beet. Columnar shadow represents available N and P contents in the rhizosphere.

The spatial variations in available N and Olsen-P contents in the root-soil system of suaeda and sugar beet are shown in Figure 3. The available N content was generally higher in the 0.7% salt level than in the salt level for suaeda, but not significantly different between the two treatments for sugar beet (Figures 3A,B). In the 0.7% salt level, the available N content in the rhizosphere was lower for suaeda and higher for sugar beet. The Olsen-P content decreased by 0.50 g·kg^−1^ and 0.99 g·kg^−1^ from the rhizosphere to the non-rhizosphere in suaeda and sugar beet under 2.7% salt level. The Olsen-P concentration in the rhizosphere was higher than in the non-rhizosphere for sugar beet under 0.7% salt level (Figures 3C,D).

### The Na^+^, Cl^−^, K^+^, and Mg^2+^ Contents

**Figure 4:**
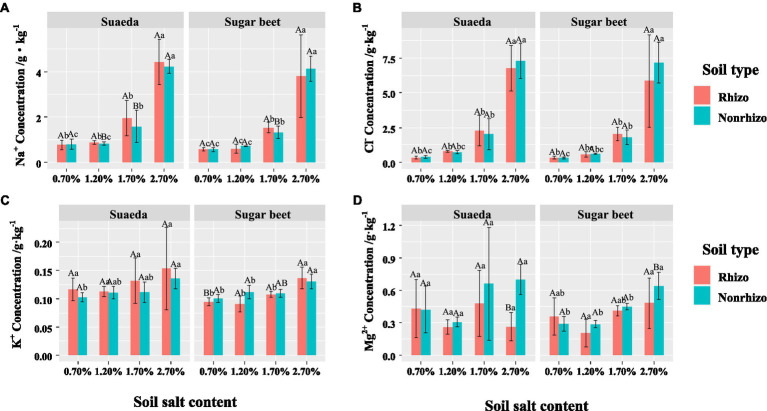
Na^+^ **(A)**, Cl^−^ **(B)**, K^+^ **(C)**, and Mg^2+^ **(D)** in the rhizosphere and non-rhizosphere of suaeda and sugar beet. Capital letters indicate the significant difference between species. Lower case letters indicate significant differences of four salt levels. Rhizo and Nonrhizo indicate the rhizosphere (0–3 mm) and non-rhizosphere (3–50 mm), respectively.

**Figure 5:**
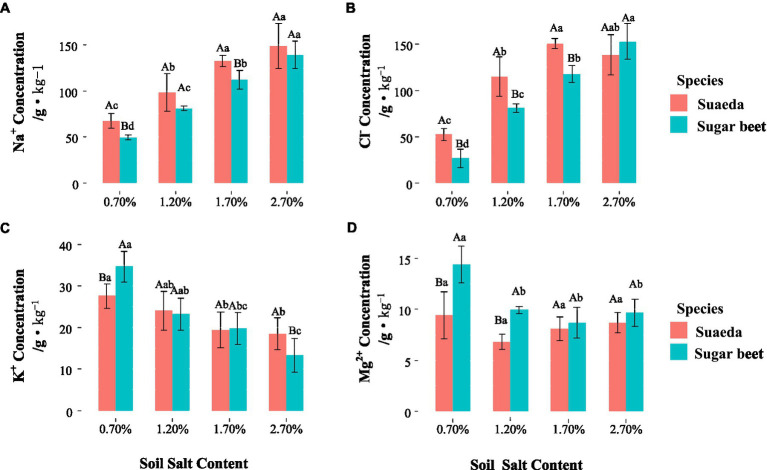
Na^+^ **(A)**, Cl^−^ **(B)**, K^+^ **(C),** and Mg^2+^ **(D)** in the shoot of suaeda and sugar beet. Capital letters indicate the significant difference between species. Lower case letters indicate significant differences of four salt levels.

For suaeda, the Na^+^ and K^+^ concentrations in the rhizosphere were comparatively higher than that in the non-rhizosphere under salinity, while for sugar beet the performance was not obvious (Figures 4A,C). The differegher than that in sugar beet, and it showed an increasing trend with salt level the two species (Figures 5A,B). The K^+^ content in suaeda and sugar beet displayed a decreasing trend, and it preformed higher in suaeda than that in sugar beet under 2.7% salt level (Figure 5C). The Mg^2+^ content in suaeda was significantly lower than that in sugar beet under 0.7 and 1.2% salt levels, and it always expressed highest in sugar beet under 0.7% salt level, but not obvious in suaeda (Figure 5D).

## Discussion

### Species-Dependent Growth Responses to Salt Stress

In the present study, we detected differences in the effects of salt stress on growth and root morphology between suaeda and beet. Different responses were detected between the two species and among the salt treatments (Figure 1). The results showed that the biomass of suaeda was highest in the 1.2% salt level, while that of sugar beet gradually decreased with increasing NaCl concentrations in soil. Our results are similar to those of previsssous studies in which suaeda biomass was higher in soils containing 100–200 mM NaCl than in soils containing lower concentrations of NaCl (Nobtitoshi and Iloru, 1991; Belkheiri and Mulas, 2013; Zouhaier et al., 2015; Feng et al., 2017; Ma et al., 2019). This would be explained by ion compartmentalization that vacuoles accumulate a large amount of sodiusssm ions so as to decrease water potentials and weaken salt stress (Song, 2009). Guo et al. (2020) explored the brown seeds of *Suaeda salsa* from NaCl-treated plants and discovered a higher germination rate under high NaCl conditions, even at 400 mM NaCl. This suggested that Na^+^ can be an essential factor for development of *Suaeda salsa* in life history. Leng et al. (2019) always found sodium (Na^+^) is the critical factor leading to the positive halotropism of the halophyte *Limonium bicolor*. The Na^+^ and Cl^−^ concentratiosssns in the shoot of suaeda were displayed higher than that in sugar beet (Figure 4), which always suggests that suaeda has more adaptive capacity to salt stress. Moreover, under high salinity, the K^+^ and Na^+^ might exist competition and induce K^+^ deficiency in th4 rhizosphere, and depolarization of the plasma membrane also stimulates the K^+^ outward rectifying channels to mediate the efflux of K^+^ and the influx of Na^+^, which could be causing the decrement of K^+^ concentration in the shoot of suaeda (Behdad et al., 2021). The higher Mg^2+^ content in sugar beet would be benefit for activating chlorophyll and enzyme to strengthen salt stress (Guo et al., 2020). In present study, the salinity levels for maximum growth and the upper limits for survival were somewhat inconsistent with other studies, which might be influenced by the soil moisture content, culture method, and plant species (Liu et al., 2018; Xu et al., 2020). The shoot/root ratio of suaeda was significantly higher than that of sugar beet, indicating that there might be an underlying mechanism for the smaller roots of halophytes to satisfy the needs of above-ground growth. For suaeda, the shoot/root ratio was significantly lower under high salinity than under lower salinity (*p* < 0.05), suggesting that this halophyte might reinforce certain root traits to promote salt resistance of below-ground parts. An adjustment in biomass allocation as an adaptive strategy to salt stress would ensure the growth of halophytes (Yuan et al., 2010; González-Orenga et al., 2020).

### Changes in Root Morphology Under Salt Stress

Roots are subject to direct damage by saline-alkali soil. Thus, changes in root morphology (root length and root diameter) and physiological manifestations are the most direct adaptive strategies for the effective absorption and utilization of soil nutrients under adverse conditions (Bates and Lynch, 1996; Mao et al., 2016; Alharby et al., 2018; Yin et al., 2021). The responses of root morphology to salt stress differed between suaeda and sugar beet (Figure 1). For suaeda, the higher root biomass and longer root length in the 1.2% salt level suggested that root length is an important factor in the adaptation to salt stress. Thus, a certain amount of salt in soil promotes root formation in halophytes, although excessive salt concentrations can have an inhibitory effect, as reported in other studies (Xiong et al., 2020; Yin et al., 2021). Xu et al. (2021) cloned the *Limonium bicolor* homolog of *Arabidopsis thaliana* and determined 100 mM NaCl-enhanced germination and root lengths. Glycophytes have also been reported to increase their root mass to increase nutrient uptake from infertile soils (Li et al., 2014; González-Orenga et al., 2020). The increase in specific root length may reflect a greater ability to absorb salt. Changes in root diameter in response to salt levels differed between the two species in this study, the diameter decreased with increasing salinity and suaeda, but this did not occur in sugar beet. This implied that suaeda increases the specific surface area of roots to increase nutrient and salt uptake under high salinity, as reported in other studies (Yang et al., 2016; Regine et al., 2018).

### Variations in Rhizosphere Properties

We detected significant differences in rhizosphere processes between the halophyte and the non-halophyte in this study. The EC in the rhizosphere was higher for suaeda, indicating that the roots of suaeda had a stronger ability to absorb salt. This is also explained that the Na^+^ and K^+^ in the rhizosphere of suaeda displayed relatively higher than that in the non-rhizosphere (Figure 4). However, the pH in the rhizosphere was higher for suaeda than in the non-rhizosphere, different from the situation in glycophytes reported in previous studies (Lyu et al., 2016; Ramzani et al., 2017). Generally, H^+^ secretion would lead to a decrease in the pH of the rhizosphere, thereby altering the physicochemical and biological processes in the rhizosphere under adverse conditions (Gahoonia et al., 2000). The variations in pH might be because CO_3_^2−^ and HCO_3_^−^ accumulated in the root zone of suaeda, as reported elsewhere (Yi et al., 2008). A critical aspect of halophytes is that they can adapt to saline conditions to ensure their survival (Xiong et al., 2020). Analyses of nutrient contents in the rhizosphere suggested that the ability to assimilate and activate soil Olsen-P in the root zone was enhanced under salt stress in sugar beet, whereas the ability to uptake available N was increased in suaeda (Figure 3). This suggests that sugar beet relies on root activities to increase the absorption of phosphate for plant growth under adverse conditions, as was found in previous studies (Gahoonia et al., 2000; Radhakrishnan and Baek, 2017; Chaudhary et al., 2018). For suaeda, the available P content was higher in the rhizosphere than in the non-rhizosphere, which indicated that H^+^ secretion activated soil nutrients to facilitate microbial activity and absorption by the roots (Zou et al., 2018). In our study, the soil pH decreased with increasing salt content (Figure 3), mainly because salinity stress results in the discharge of H^+^ (Yi et al., 2008). Higher salt concentrations promote the absorption of positive ions by halophytes so that the rhizosphere soil becomes enriched with anions and secreted H^+^ (Ben Hassine et al., 2009). Both salinity and pH affect bacterial communities, extracellular enzyme, and thus nutrient availability in saline soil (Lauber et al., 2009; Zhao et al., 2018). In addition, the roots of halophytes can secrete metabolites into the rhizosphere to stimulate microorganisms and regulate nutrient availability, ultimately enhancing plant growth and salt tolerance (Badri and Vivanco, 2009; Sashidhar and Podile, 2010; Jha et al., 2011; Yuan et al., 2017).

## Conclusion

In this study, moderate salinity positively affected the biomass of suaeda, but negatively affected that of sugar beet. A certain salt always promoted root elongation of suaeda, but high salinity decreased the root diameter. For suaeda, high salinity increased the pH, EC, and Na^+^ content to higher levels in the rhizosphere than in the non-rhizosphere. For sugar beet, high salinity resulted in lower pH and higher available N and Olsen-P contents in the rhizosphere. This suggested that the two species use different strategies to absorb nutrients under high salinity: that is, suaeda acquires more nutrients by increasing root formation, whereas sugar beet adjusts rhizosphere processes. This study provides useful information for better figuring out the salt tolerance mechanism and strategy in nutrient uptake of halophyte under high salinity based on root morphological and rhizospheric traits. The further researches would be to detect the root system architecture, the influence of root secretion on the rhizosphere properties, and interactions among root-microorganism-soil under salinity in the future.

## Data Availability Statement

The original contributions presented in the study are included in the article/supplementary material, further inquiries can be directed to the corresponding authors.

## Author Contributions

SW: data curation, investigation, methodology, software, and writing – original draft. ZZ: conceptualization, formal analysis, investigation, methodology, supervision, and validation. SG: data curation, investigation, methodology, and resources – equal. BP: data curation, formal analysis, investigation, methodology, and software. KZ: conceptualization, investigation, methodology, and resources. MH: data curation, investigation, methodology, and supervision. WM: conceptualization, funding acquisition, methodology, project administration, validation – lead, visualization, and writing – review and editing. CT: funding acquisition, methodology, project administration, validation, visualization, and writing – review and editing.

### Conflict of Interest

The authors declare that the research was conducted in the absence of any commercial or financial relationships that could be construed as a potential conflict of interest.

## Abbreviations

N: nitrogen
P: phosphorus
Olsen-P: available phosphorus
pH: pondus hydrogenii
EC: electrical conductivity
